# Case report: Occult lymph nodal metastasis in sub-centimeter lung cancer: a report of seven cases and review of literature

**DOI:** 10.3389/fonc.2025.1582033

**Published:** 2025-07-14

**Authors:** Xiao Wang, Yingxin Li, Xiaoxuan Shi, Zhengcheng Liu

**Affiliations:** Department of Thoracic Surgery, Nanjing Drum Tower Hospital, Affiliated Hospital of Medical School, Nanjing University, Nanjing, Jiangsu, China

**Keywords:** sub-centimeter lung cancer, lymph node metastasis, pathology, radiology, case report

## Abstract

**Background:**

Occult lymph node metastasis (OLNM) after surgery for stage IA sub-centimeter lung cancer is an extremely rare clinical situation, with few reported cases in the literature. Further investigation is warranted into the clinical characteristics of patients and the radiological features of lung nodules. Here, we aim to provide a comprehensive insight into this tumor type.

**Methods:**

We present cases that sub-centimeter non-small cell lung cancer (NSCLC) patients who underwent surgery at our center and were found to have OLNM in postoperative pathology. This is the first time such cases have been reported. We reviewed the existing literature on this rare event and collected detailed clinical, histopathological, and radiological data for analysis.

**Results:**

A total of 7 sub-centimeter lung cancer patients were diagnosed with OLNM based on routine pathology, including 1 male and 6 females, with an average age of 58.7 ± 9.3 years. All patients had no clinical symptoms and were found by physical examination, with follow-up durations ranging from 1 week to 3 years. Among these patients, 4 exhibited mediastinal lymph node metastasis. Preoperative chest CT in all patients showed pure-solid nodules (PSN), and 6 patients changed the surgical procedure based on intraoperative frozen sections results. Routine pathology revealed that all adenocarcinoma patients presented high-risk factors for recurrence, including visceral pleural invasion (VPI), spread through air spaces (STAS), vascular invasion, and poorly differentiated tumor.

**Conclusion:**

Sub-centimeter lung cancers with radiological feature of PSN are at risk for lymph node metastasis. Surgeons should pay close attention to intraoperative rapid pathology results to prevent inadequate surgical treatment. Adenocarcinoma patients often present with one or more high-risk factors for recurrence, active adjuvant treatment and follow-up are necessary.

## Introduction

Sub-centimeter non-small cell lung cancer (NSCLC) generally exhibits low malignancy, with minimally invasive adenocarcinoma (MIA) being predominant, and occurrences of occult lymph node metastasis (OLNM) are rare (1). OLNM is defined as lymph node metastasis that is undetected during clinical evaluation but discovered incidentally upon pathological analysis (2). To our knowledge, this is the first comprehensive presentation of the pathological and radiological characteristics of these tumors.

## Methods

We report the comprehensive diagnosis and treatment process of these cases to provide insights into this rare condition.

Additionally, we conducted a literature search in PubMed and Web of Science to identify all relevant articles published in English. We aim to explain the underlying reasons for these phenomena and suggest improvements for our treatment strategies in future clinical practice. The specific characteristics of all cases are detailed in **Table 1**.

**Table 1:** Radiological features of seven cases.

Case	Type	Diameter	Mean CT value	Location	Lobulation	Spiculation
1	PSN	10*9mm	124.66Hu	RLL	Present	Present
2	PSN	10*9mm	31.62Hu	LUL	Present	Absent
3	PSN	10*9mm	29.54Hu	LLL	Present	Absent
4	PSN	10*8mm	76.86Hu	LLL	Present	Present
5	PSN	10*7mm	20.82Hu	RLL	Present	Present
6	PSN	7*7mm	184.88Hu	RUL	Present	Absent
7	PSN	8*6mm	-139.79Hu	LLL	Absent	Absent

PSN, Pure-solid nodule; LLL, Left Lower Lobe; LUL, Left Upper Lobe; RLL, Right Lower Lobe; RUL, Right Upper Lobe.

## Case report

This study comprises seven cases, including one male and six females, with an average age of 58.7 ± 9.3 years. All patients were asymptomatic and discovered incidentally by physical examinations. The follow-up period ranged from one week to three years, and none of the patients received neoadjuvant therapy before surgery. Preoperative chest CT scans revealed that four lesions were located on the left side and three on the right. The pulmonary nodule types of all patients exhibited pure-solid nodules (PSN), with a mean CT value of 46.9 ± 94.2 Hu. Six patients’ radiology demonstrated lobulated signs, while three had spiculated signs. The radiological features of the 7 cases are detailed in **Table 1**.

All patients underwent video-assisted thoracic surgery (VATS) without CT-guided preoperative localization. The procedures were performed using either a uniportal or double-port approach; the uniportal technique involved a 3cm incision from the anterior axillary line to the midaxillary line between the fourth or fifth intercostal, while the double-port technique included an additional Trocar incision at the seventh intercostal. During surgery, six patients with peripheral nodules underwent wedge resection with margins ≥2cm, awaiting intraoperative frozen pathology results. One patient with a central nodule, due to the deep location of the lesion and after discussion with the patient and family, underwent lobectomy directly. As the rapid intraoperative pathology indicated invasive adenocarcinoma, so systematic lymphadenectomy was performed. Among the six patients who underwent wedge resection, five were diagnosed with invasive adenocarcinoma, and one was found to have a lymphohematopoietic malignancy with the group 11 lymph nodes metastasis. Ultimately, all six patients had their resection extended to include lobectomy or segmentectomy, along with lymph node dissection. The average operation time was 112.9 ± 27.9 minutes, and thoracic drainage tube was placed through the single operation port or Trocar port for negative pressure drainage.

## Result

All surgeries in this cohort were successful, with no significant intraoperative complications or perioperative mortality. The drainage tubes were left in place for 2 to 4 days (average 2.8 days), and the length of stay ranged from 4 to 7 days (average 5 days). To At the two-week postoperative follow-up, all patients showed good recovery with no occurrences of pleural effusion or pneumothorax.

Routine postoperative pathology revealed that five patients had invasive non-mucinous adenocarcinoma (INMA), one had invasive mucinous adenocarcinoma (IMA), and one had lymphoepithelial carcinoma (IEC). Three patients presented with hilar lymph node metastasis, while four had mediastinal lymph node metastasis, including one case with skip metastasis (only positive in the group 7). All adenocarcinoma patients exhibited one or more high-risk factors for recurrence, including visceral pleural invasion (VPI), spread through air spaces (STAS), vascular invasion (VI), and tumor poorly differentiated status (**Table 2**).

**Table 2:** Clinical characteristics of seven cases.

Case	Gender	Pathological diagnosis	Surgical procedure	pN stage	STAS	VI	VPI	Differentiation
1	Female	INMA	WR→LE	N2	Negative	Negative	Positive	Moderately
2	Female	LEC	SE→LE	N1	Negative	Negative	Negative	/
3	Male	IMA	WR→LE	N1	Positive	Positive	Positive	/
4	Female	INMA	WR→LE	N2	Positive	Negative	Negative	Moderately
5	Female	INMA	LE	N2	Negative	Negative	Negative	Poorly
6	Female	INMA	WR→LE	N2	Positive	Negative	Negative	Poorly
7	Female	INMA	WR→SE	N1	Positive	Negative	Negative	Poorly

IMA, Invasive mucinous adenocarcinoma; INMA, Invasive non-mucinous adenocarcinoma; LE, Lobectomy; LEC, Lymphoepithelial carcinoma; PSN, Pure-solid nodule; SE, Segmentectomy; STAS, Spread through air spaces; VI, Vascular invasion; VPI, Visceral pleural invasion; WR, Wedge resection.

## Discussion

According to the 2025 report from the American Cancer Society, lung cancer remains the leading cause of cancer-related mortality worldwide (3). The standard surgical treatment for early-stage lung cancer is still anatomical lobectomy. However, with the publication of clinical trials such as JCOG0802, JCOG0804, and CALGB140503, sublobar resection has emerged as a common surgical approach for early-stage lung cancer, gaining acceptance among thoracic surgeons and lung cancer patients (4–6). The widespread use of low-dose computed tomography and increased awareness of physical examination, have led to a rising number of sub-centimeter lung cancer cases, defined as malignant tumors with a maximum diameter of ≤1 cm, which generally exhibit low malignancy. Sublobar resection has a long-term effect that is not inferior to lobectomy, and can preserve the lung function of patients to the maximum extent. However, some sub-centimeter lung cancers present high-risk factors that adversely affect patient prognosis and may even lead to OLNM, complicating surgical strategy formulation and clinician-patient communication. Current research on this topic remains insufficient, making it particularly important to retrospectively analyze such high-risk nodules to enhance understanding.

We conducted a retrospective analysis of 7 patients who underwent lung cancer resection at our hospital, with postoperative pathology indicating sub-centimeter lung cancer with OLNM. The average age of the patients was 58 years, lower than the global average age of 65 for lung cancer (7). Preoperative radiology in all patients showed PSN. For patients with peripheral nodules, the surgical approach was modified based on intraoperative frozen pathology results, leading to an expanded resection. In terms of routine pathology, all adenocarcinoma patients presented with one or more risk factors for recurrence.

Based on the density on CT, lung nodules can be classified into ground glass nodules, mixed ground glass nodules and PSN. PSN refers to round or oval areas of increased density in the lung, where solid components are detected in both lung and mediastinal windows, obscuring the bronchial and vascular structures within (8). The presence of solid components in nodules is an independent risk factor for lymph node metastasis, with solid components generally indicating more aggressive disease compared to ground glass nodules of similar diameter. Yoon et al. studied the radiological characteristics of non-small cell lung cancer (NSCLC) with OLNM, finding that endobronchial tumors had the highest risk of N1 OLNM (9). The risk of OLNM was greater for PSN compared to mixed ground glass nodules, and for mixed ground glass nodules with >50% solid components, spiculated margin and peri-tumoral ground glass opacity were significant predictors of OLNM. Kawamoto et al. reported that the distance between the tumor, hilum of lung, and visceral pleura on CT could serve as an independent predictor of OLNM in lung cancer (10). Additionally, preoperative serum carcinoembryonic antigen (CEA) levels, programmed death ligand-1 (PD-L1) expression, maximum standardized uptake value (SUVmax) on PET-CT, and epidermal growth factor receptor (EGFR) gene mutations have all been associated with OLNM (11–13). In this study, only 3 PSN patients exhibited spiculated features, and the radiological characteristics surrounding tumors were poor predictors of OLNM in sub-centimeter lung cancer, likely due to the small nodule diameter.

Pulmonary infections, benign lesions, and malignant tumors can all present as nodules or shadows on CT. Intraoperative frozen pathology provides vital evidence for precise surgical treatment of lung cancer. Compared with preoperative puncture, bronchoscopy and serological examination, intraoperative frozen pathology offers higher accuracy, guiding surgical decisions and preventing undertreatment or overtreatment. The malignancy probability of nodules presenting as PSN on CT is lower than that of ground glass nodules and mixed ground glass nodules. For peripheral PSN, performing lung wedge resection can prevent excessive resection due to benign nodules, while the excision extension can be warranted for highly malignant nodules. Previously, it was thought that sub-centimeter NSCLC had no risk of lymph node metastasis. However, recent clinical practice has prompted discussions about the necessity of lymph node evaluation for small-diameter lung cancers, with ongoing debates about the optimal assessment methods (8, 14). A study by Dezube et al. found that 89.2% of sub-centimeter lung cancer cases had insufficient lymph node sampling, potentially leading to underestimated pathological staging and missed adjuvant therapy (15). Tan et al. found that the fragmentation of lymph nodes during surgery would lead to a potential miscalculation of the number of lymph nodes (16). In addition, Nobel et al. pointed out that about 15% of patients with clinical stage IA NSCLC accompanied by OLNM had mediastinal lymph node metastasis without hilar lymph node metastasis, indicating skip metastasis (17). Therefore, hilar lymph node sampling alone cannot identify all OLNM cases; mediastinal lymph node examination should be performed for invasive cancer patients. Our study indicates that even lung cancer with small tumor diameter can exhibit mediastinal lymph node metastasis, including skip metastasis. In clinical practice, for patients with intraoperative frozen pathology suggesting invasive cancer, lymph node dissection should be conducted according to established guidelines.

The National Comprehensive Cancer Network (NCCN) guidelines specify high-risk factors for postoperative recurrence in lung cancer patients, including vascular invasion, visceral pleura invasion, poorly differentiated tumors, sublobar resections, and unknown lymph node status (18). Fick et al. found that the 5-year cumulative recurrence rate of lung cancers with these high-risk features was significantly higher than that of tumors without these features (30% *vs* 4%, *P*<0.001) (19). Current guidelines recommend postoperative adjuvant therapy for patients with stage IB and above with high risk factors for postoperative recurrence. For lung cancer patients with OLNM, postoperative adjuvant therapy is necessary. Including platinum-based adjuvant chemotherapy. Patients with EGFR-sensitive gene mutations can receive Osimertinib or Icotinib as adjuvant targeted therapy. For patients with negative driver genes, if PD-L1 expression is positive, Atezolizumab adjuvant therapy can be administered after platinum-based chemotherapy (18, 20). In terms of surgical strategy, tumor location and adequate margins are crucial for performing sublobar resections. The impact of lobectomy versus sublobar resection on the prognosis of OLNM patients remains unclear (21, 22). Liou et al. identified that, compared to the surgical procedure, margin status and postoperative adjuvant therapy are key determinants of overall survival in OLNM patients (22). We propose that there may be interrelationships among the aforementioned high-risk factors, and further investigation into their collinearity and causal relationships are worthy of further study.

Based on the case report and literature review, sub-centimeter lung cancers presenting as PSN is at risk of OLNM. It is essential to conduct thorough preoperative evaluations and maintain effective communication between doctors and patients. Regular follow-up and reexamination of chest CT should be conducted. If necessary, PET-CT examination or biopsy examination can be performed to detect signs of cancer dissemination and confirm lung cancer. During surgery, attention should be paid to the frozen pathology to prevent inadequate surgical treatment. Lung adenocarcinomas often exhibit one or more high-risk recurrence factors, active adjuvant treatment and follow-up should be carried out.

## Data Availability

The raw data supporting the conclusions of this article will be made available by the authors, without undue reservation.
